# Utility of Optical Genome Mapping for Accurate Detection and Fine-Mapping of Structural Variants in Elusive Rare Diseases

**DOI:** 10.3390/ijms26031244

**Published:** 2025-01-31

**Authors:** Carmen Orellana, Monica Rosello, Amparo Sanchis, Laia Pedrola, Carla Martín-Grau, Alba Gabaldón-Albero, Maria Leonor Senent, Esperanza Such, Cristian García-Ruiz, Gayane Avetisyan, Francisco Martínez

**Affiliations:** 1 Genetics Unit, Hospital Universitario y Politécnico La Fe, 46026 Valencia, Spain; 2 Traslational Genetics Research Group, Instituto de Investigación Sanitaria La Fe (IIS La Fe), 46026 Valencia, Spain; 3 Pediatrics Service, Hospital Universitario Doctor Peset, 46017 Valencia, Spain; 4 Hematology and Hemotherapy Service, Hospital Universitario y Politécnico La Fe, 46026 Valencia, Spain; 5 Hematology Research Group, Instituto de Investigación Sanitaria La Fe (IIS La Fe), 46026 Valencia, Spain; 6 Centro de Investigación Biomédica en Red en Cáncer (CIBERONC), Instituto de Salud Carlos III, 28029 Madrid, Spain

**Keywords:** optical genome mapping, structural variants, balanced translocation, enhancer–promoter interactions, congenital malformations, *IHH*, BCL11A

## Abstract

Rare diseases (RDs) often have a genetic basis, yet conventional diagnostic techniques fail to identify causative genetic variations in up to 50% of cases. Structural variants (SVs), including balanced rearrangements, frequently evade detection by karyotyping, microarray, and exome sequencing. The present study utilized optical genome mapping (OGM) to investigate two patients with RDs whose genetic etiology remained unresolved despite prior genomic analyses. Patient 1 exhibited a balanced reciprocal translocation disrupting the *BCL11A* gene, associated with Dias-Logan syndrome. Patient 2 had a mosaic 682 kb deletion near the *IHH* gene, causing ectopic enhancer–promoter interactions and polydactyly, mirroring phenotypes observed in mouse models and similar human cases. These findings highlight OGM’s efficacy in identifying complex SVs and underline novel pathogenic mechanisms in rare genetic disorders. Consequently, the incorporation of OGM into routine diagnostic procedures will enhance genetic diagnosis, discover new syndromes of currently unknown cause, and eventually improve the clinical management of numerous patients with rare diseases.

## 1. Introduction

Rare diseases (RDs) encompass conditions frequently associated with genetic factors. In the last decade, array analysis and whole exome sequencing have become broadly used for the genetic diagnosis of individuals with RDs. However, the diagnostic yield of these approaches, even when combined, only reaches at most 30% to 50% depending on the clinical cohort and the strategy used [1]. The inability to reach an etiological diagnosis causes emotional stress for both the family and clinicians, hindering appropriate familiar genetic counseling. It is assumed that this limited diagnostic yield is partly explained by the fact that some causal genetic variations remain elusive to these techniques. Disruption in critical genes or regulatory regions may underlie the genetic etiology of these RDs [2,3]. Balanced rearrangements and certain structural variants (SVs) often remain undetected by many genomic techniques, including karyotyping, microarray, and exome sequencing [4]. For instance, balanced reciprocal translocations are relatively common, with an estimated frequency of 0.25% [5]. While most balanced translocations are not a direct cause of disease, healthy carriers are at risk of having children with unbalanced translocations, which, in many cases, result in non-viable conditions or lead to severe phenotypic consequences in the offspring. However, in some cases, the disruption of a critical gene at the breakpoint regions can lead to significant phenotypic effects. Therefore, at least in the de novo rearrangements, it is crucial to define the breakpoints involved in structural rearrangements to determine if the coding sequence of a gene has been disrupted or if the critical position of remote regulatory elements—such as enhancers, repressors, or insulator sequences—has been altered, potentially deregulating the proper expression of a neighboring gene.

Optical genome mapping (OGM) is a powerful technique first described in 2009, designed to visualize and analyze the structure of entire genome at high resolution. OGM allows the detection of large-scale structural variations in DNA, as well as mosaic alterations that may be overlooked by other genetic analysis techniques. We performed OGM in two patients who remained genetically unsolved after prior genomic approaches failed to identify pathogenic variants. This study aimed to take a more comprehensive view into the genomic landscape of rare variants implicated in RDs using OGM. This technique has the potential to reveal novel mechanisms involving complex SVs that are often missed by standard genetic techniques.

## 2. Detailed Case Description

### 2.1. Brief Description of Patient 1

**Figure 1:**
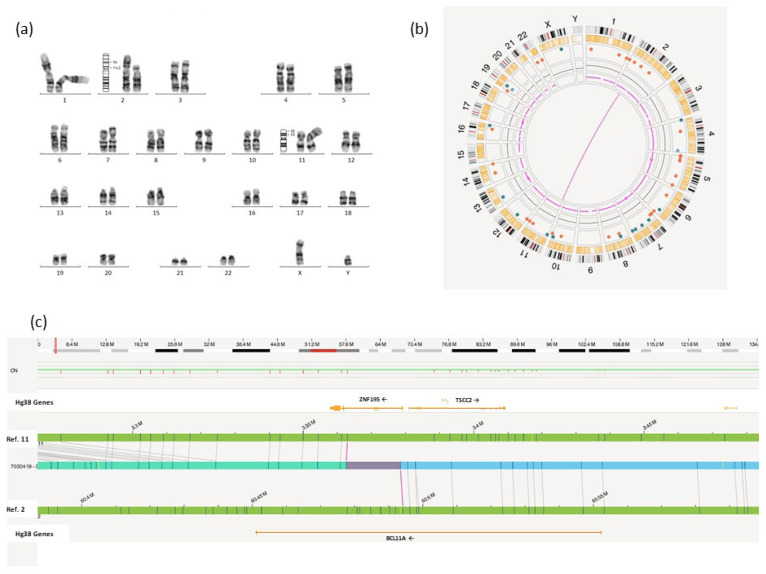
Genetic analyses performed on Patient 1. (**a**) G-banding karyotyping and optical genome mapping results. The ideograms next to chromosomes 2 and 11 indicate the location of two breakpoints: those initially proposed from the G-banding sequence t(2;11)(p11.2;p13) and those confirmed as the true breakpoints by optical genome mapping t(2;11)(p16.1;p15.4). (**b**) Circus plot shows a pink line connecting chromosome 2 and chromosome 11, which represents the balanced translocation between them. (**c**) The genome map view illustrates the balanced translocation (ogm[GRCh38]t(2;11)(p16.1;p15.4)(60494146;3362963)). The breakpoint on chromosome 2 is located within the *BCL11A* gene.

Patient 1 is a 5-year-old boy, the only child of a single-parent family, with no family history of neurodevelopmental disorders. The pregnancy was the result of in vitro fertilization by intracytoplasmic injection of donor sperm. During the pregnancy, mild polyhydramnios and dilatation of the right renal pelvis were detected. The patient was born at 41 weeks of gestation by cesarean section showing macrosomia, with birth weight of 4.540 g, length of 55 cm, head circumference of 37 cm, all three measurements exceeding the 97th percentile, according to International Standards for Newborn Weight [6]. Clinical findings also included a prominent forehead, bilateral strabismus, flattened nasal bridge, hydronephrosis of the right kidney, and dysplasia of the left kidney. At the follow-up, significant psychomotor and language delay became evident, accompanied thereafter by moderate intellectual disability. Cerebral MRI study showed no pathological findings. Peripheral blood karyotyping revealed a reciprocal translocation in the patient, which was not detected in the blood cells of either the mother or the sperm donor (46,XY,t(2;11)(p11.2;p13)dn) (Figure 1a). Array comparative genomic hybridization (CGH-array) analysis showed no abnormalities, indicating a balanced translocation without loss or gain of genetic material (array CGH 180 K Agilent Technologies, Santa Clara, CA, USA). Clinical exome sequencing was performed and did not reveal any significant findings (New Focused Exome v2; Agilent Technologies, Santa Clara, CA, USA). OGM analysis confirmed the translocation between chromosomes 2 and 11, redefining the breakpoint regions more precisely, from 46,XY,t(2;11)(p11.2;p13)—as identified in Karyotyping—to 46,XY,t(2;11)(p16.1;p15.4) (Figure 1b). While the breakpoint region identified on chromosome 11 did not encompass any gene with a clearly known function, this chromosomal rearrangement led to the disruption of the *BCL11A* gene located on chromosome 2. The sites surrounding the translocation breakpoint region are located at positions chr2:60,494,146, in the large intron 2 of the *BCL11A* gene, and chr11:3,362,963, in the intron 3 of the *ZNF195* gene, respectively (ogm[GRCh38] t(2;11)(p16.1;p15.4)(60494146;3362963)) (Figure 1c). However, the uncertainty interval in chromosome 11 extends to intron 1 of the *ZNF195* gene. No additional SVs with clinical relevance were identified.

Given the established role of the *BCL11A* gene in hemoglobin switching and fetal hemoglobin (HbF) silencing [7], hemoglobin electrophoresis and quantification were performed. The analysis revealed elevated HbF at 5% (normal < 0.5%) without other hematological abnormalities (Hb: 12.3 g/dL).

Combining cytogenetics, molecular analyses, clinical data, and HbF monitoring, this de novo translocation was considered pathogenic, suggesting Dias-Logan syndrome in the patient caused by *BCL11A* haploinsufficency.

### 2.2. Brief Description of Patient 2

Patient 2 is the third child of healthy non-consanguineous parents. She was born in 1983 with a polymalformative syndrome characterized by agenesis of the corpus callosum, minor facial dysmorphism, and complex polysyndactyly (mirror image of hands and feet) with a total of 33 fingers and toes, without thumbs, multiple joint abnormalities, and femoral shortening. A detailed description of the clinical phenotype was published in 1985 suggesting a new type of acrocallosal syndrome [8]. She is currently 41 years old, and the facial dysmorphism has become more pronounced over the years, including broad and prominent forehead, down slanting palpebral fissures, broad nasal root, short nose, long flattened philtrum, small mandible, and low-set dysplastic and posteriorly rotated ears. She has rhizomelic shortening of the lower limbs, normal muscle tone and mobility in her limbs, but is unable to stand or walk. She is severely intellectually disabled and is dependent for hygiene and feeding. Despite this, she demonstrates social interaction and limited language consisting of single words and simple phrases with echolalia. Behavioral problems include tantrums, phobias, and night terrors. Over the years, many genetic studies have been carried out on this patient to find the cause of the malformations. Cytogenetic analysis, including high resolution karyotyping and genomic array studies with two different approaches—a high-density oligo array (Affymetrix CytoScan HD SNP array, Life Technologies, Carlsbad, CA, USA) and CGH-array 160 K (Nimgenetics)—did not reveal any genetic abnormalities in the patient. Similarly, whole exome sequencing only identified a variant of unknown clinical significance, NM_001164405.2:c.305 C > A; *p*.(Ser102 Tyr) in the *BHLHA9* gene, which was ruled out as causative after familial segregation analysis.

**Figure 2:**
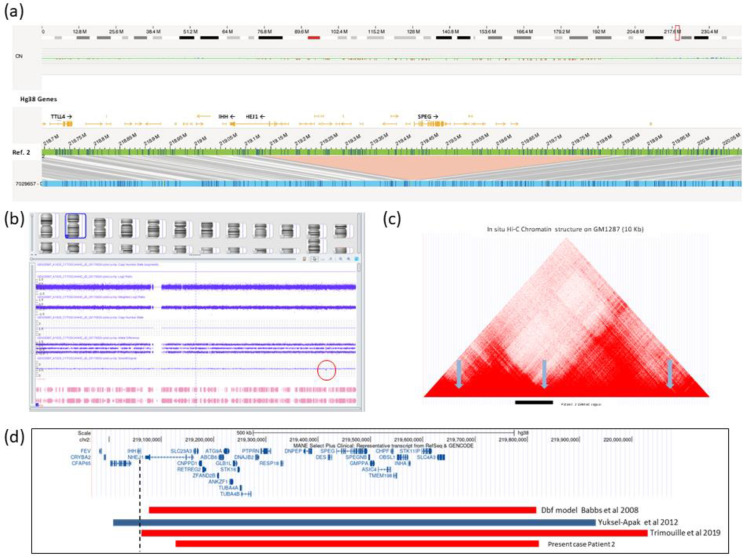
Genetic analyses performed on Patient 2. (**a**) Optical genome mapping results. The genome map view shows a heterozygous 682,108 bp deletion overlapping 30 genes, with the nearest non-overlap gene being *IHH* (ogm[GRCh38] 2q35(219132322_219826404)x1). The allele frequency of 0.27 suggests a mosaic condition. (**b**) Results from the high-resolution genomic array (Affymetrix CytoScan HD SNP array). Image of chromosome 2 shows only a slight decrease in probes in the deletion region, without reaching significant values. (**c**) Hi-C data from Rao et al. [9] (GM12878 cell line, 10 kb resolution). This region encompasses several topologically associated domains (TADs), with the predicted TAD unions marked by blue arrows. The horizontal black bar at the bottom represents the deleted region in Patient 2, showing that this deletion could eliminate one of the TAD junctions or insulators in that region. The image was obtained from the UCSC Genome Browser on Human (GRCh37/hg19) with a converted genomic coordinate (chr2:219997044-220691125). (**d**) Genomic overview of the deleted region on chromosome 2q35 shows the genomic regions affected in other published cases with a similar phenotype [10,11,12]. The red bars represent deletions, and the blue bar represents duplication. The vertical dashed line indicates the 5′ end of the *IHH* gene, showing that only the duplication described by Yuksel-Apak [10] directly affects the *IHH* gene, while the deletions have breakpoints that impact different regions of the *NHEJ1* gene, located distally but very close to *IHH*.

OGM study identified a mosaic heterozygous 682 kb deletion of the chromosomal region 2q35 (ogm[GRCh38] 2q35(219132322_219826404) x1), involving 30 different genes (*NHEJ1, SLC23A3, CNPPD1, RETREG2, ZFAND2B, ABCB6, ATG9A, ANKZF1, GLB1L, STK16, TUBA4A, TUBA4B, DNAJB2, PTPRN, MIR153-1, RESP18, DNPEP, DNPEP-AS1, DES, SPEG, SPEGNB, GMPPA, ASIC4, CHPF, TMEM198, MIR3132, OBSL1, INHA, STK11IP*, and *SLC4A3*) (Figure 2a). This mosaic alteration, with a variant allelic frequency of 0.27, had remained undetected in all previous genetic analysis, including genomic array studies. However, a posterior visual inspection of the region in the array confirmed a slight decrease in the signal intensity for all probes in the affected region, without reaching the threshold value established by the manufacturer for variant calling (Figure 2b).

This deletion is not reported in the population control databases (DGV). It should be noted that one of the breakpoints of the deleted region is located very close to the *IHH* gene (Indian Hedgehog). This deletion was considered as pathogenic on evidence from similar deletions or duplications near the *IHH* gene that have been previously associated with a highly similar phenotype.

## 3. Discussion

This study provides a more comprehensive assessment of the power of OGM to detect SVs implicated in rare diseases, which were previously missed by standard genetic techniques such as exome sequencing and microarray study, and even if SVs are known to be present, OGM provides higher resolution of the breakpoint and allows for a deeper understanding of the underlying molecular mechanism. In Patient 1, the *BCL11A* gene was disrupted by a balanced chromosomal translocation t(2;11). The BCL11A protein is a C2H2-type zinc-finger protein associated with the BAF SWI/SNF chromatin remodeling complex [13]. This gene plays a critical role in regulating the developmental switch from gamma to beta globin, hence indirectly repressing HbF levels, as well as in brain development [14]. Haploinsufficency of this gene, whether due to heterozygous truncating mutations or missense variants in the N-terminal region, has been proved to cause Dias-Logan syndrome [OMIM #617101]. This syndrome is characterized by intellectual disability, language delay, persistence of HbF, and variable dysmorphic features, including microcephaly [7]. The intellectual developmental disorder associated to the persistence of HbF observed in our patient confirms that the translocation results in a functional impairment of the *BCL11A* gene.

On the other hand, it is highly conceivable that as a result of the chromosomal translocation, two novel fusion transcripts may be formed, which may be pathogenic through different mechanisms, including the presence of a novel polypeptide that combines functional domains from two different genes and/or a putative dominant-negative effect due to the preservation of functional domains in partially truncated proteins [15]. The genes located at the breakpoints in Patient 1 are transcribed in the same sense, and exon 3 of the *BCL11A* gene starts in the same reading frame as exons 3 and 4 of the *ZNF195* gene. On the other hand, if the breakpoint is located within intron 1, a frameshift would occur. We cannot rule out a putative contribution of one or both fusion transcripts to the phenotype, which might explain the clinical findings in our patient that have not been previously reported in Dias-Logan syndrome, namely macrosomia and renal abnormalities. Nonetheless, it remains uncertain whether these novel chimeric proteins are synthesized, whether they are not rapidly degraded in the cell, whether they are able to translocate from the cytoplasm into the nucleus, or even whether they in fact confer a pathogenic disturbance. In consequence, a possible contribution of the chimeric genes to the phenotype is highly hypothetical at this moment. In any case, the loss of exons 1 and 2 of the *BCL11A* gene (substituted by exons 1-3 of *ZNF195*) is expected to result in a loss of function, as it implies the loss of both the NuRD-interacting domain and a C2HC zinc-finger domain involved in protein–protein interaction [14]. It is worth noting that missense variants in these domains lead to a defective dimerization, localization, and transcriptional activity, clinically indistinguishable from those caused by a truncating variant [7].

OGM in Patient 2 identified a mosaic microdeletion of 682 kb in the chromosomal region 2q35, which affects 30 genes, and is located very close to the *IHH* gene. This mosaic alteration (estimated 27%) remained undetected by all previously used genetic analysis techniques. Array-based techniques, which are considered the gold standard for detecting copy number variants, are limited in their ability to identify low-grade mosaicisms, particularly in cases of small segmental aneusomies (less than 30%) [16]. In contrast, OGM is based on the identification and counting of individual DNA molecules and not on relative quantification, making it more efficient for detecting large-scale SVs. OGM demonstrates high sensitivity for low-frequency alterations, simplifies the interpretation of complex alterations, and operates independently of coverage or sequencing challenges associated with arrays and NGS. This renders OGM a valuable tool, especially for analyzing mosaics and SVs that are difficult to identify with other technologies. Although the deletion does not directly affect the *IHH* gene, it alters the genomic organization, potentially leading to ectopic interactions between enhancers and promoters, and causing aberrant gene expression patterns as previously suggested (Figure 2c). A very similar deletion in the nearby distal region of the *IHH* gene was identified in the Doublefoot (Dbf) mouse model [11]. This mutant mouse displays phenotypic features very similar to our Patient 2, including preaxial polydactyly with 6-9 triphalangeal digits on all four limbs, tibial hypoplasia, widened skull, hydrocephalus, and thickened curled tail. In humans, another comparable case was a female fetus with a microdeletion overlapping that of Patient 2, where the centromeric breakpoint is located 429 base pairs downstream of the transcription start site of the *IHH* gene [12] (Figure 2d). Clinical findings included, among other malformations, extensive polydactyly, with eight fingers on each hand with a mirror image of the right hand, seven fingers on the left foot and six fingers on the right foot with an enlarged hallux. Additionally, there is a remarkable clinical resemblance between Patient 2 and other individuals presenting features similar to acrocallosal syndrome. This individual exhibited extensive polysyndactyly of the hands and feet, craniofacial abnormalities including macrocephaly, agenesis of the corpus callosum, dysplastic and low-set ears, severe hypertelorism, and profound psychomotor delay caused by a large duplication involving the *IHH* locus [10] (Figure 2d).

Although haploinsufficiency of some of the genes contained in the deletion may partially contribute to the phenotype in the patient, none of them have been directly linked to polydactyly nor do they show constraint scores suggestive of being haploinsufficient (LOEUF < 0.3), and hence to be sensitive to heterozygous deletions [17]. Notably, the proximal breakpoint of the deletion in Patient 2 is located very close to the *IHH* gene. This gene encodes a member of the Hedgehog protein family, essential secreted signaling molecules that regulate a variety of developmental processes including growth, pattern formation and morphogenesis. The protein encoded by the *IHH* gene plays a specific role in bone growth and differentiation. Mutations in this gene are the cause of brachydactyly type A1, characterized by shortened or malformed fingers and toes, and acrocapitofemoral dysplasia. Furthermore, Lupiañez et al. [18], using CRISPR/Cas genome editing and expression studies in mouse limb tissue and patient-derived fibroblasts, demonstrated that disruption of TADs (Topologically Associated Domains) can rewire long-range regulatory architecture and result in pathogenic phenotypes. Their study revealed that distinct human limb malformations are caused by deletions, inversions or duplications altering the structure of the TAD-spanning *WNT6/IHH/EPHA4/PAX3* locus. Several disease-relevant structural changes cause ectopic interactions between promoters and non-coding DNA, and a cluster of limb enhancers normally associated with *EPHA4* is misplaced relative to TAD boundaries and drives ectopic limb expression of another gene in the locus. This rewiring occurred only when the variant disrupted a CTCF-associated boundary domain. Their findings underscore the functional importance of TADs in orchestrating gene expression via genome architecture and indicate criteria for predicting the pathogenicity of human structural variants, particularly in non-coding regions of the human genome. This robust experimental evidence has demonstrated that some SVs, depending on the size and position, may disrupt higher order genomic organization and result in pathogenic phenotypes. 

It is highly relevant that, in spite of being in somatic mosaicism, the clinical features of Patient 2 closely resemble the phenotype of the Dbf mouse model caused by a similar deletion, as well as the acrocallosal-like syndrome resulting from *IHH* duplication. In particular, the polydactyly of the female fetus reported by Trimouille et al. [12] is the same as in Patient 2, with a mirror configuration of the fingers. Therefore, we hypothesize that the main pathophysiological effect of the deletion in this patient, even in mosaicism state, is the same: an ectopic interaction between the *IHH* gene and enhancers in the chromatin domain of *EPHA4* due to a reorganization of the TAD boundaries. Our results reinforce the notion that the pathogenicity of some human disease-associated deletions results from ectopic enhancer–promoter interactions causing ectopic/dysregulated genic expression due to the elimination of annotated boundaries [19].

OGM holds great promise for improving the diagnosis of unresolved genetic disorders, especially in the identification of complex SVs or mosaic alterations that are challenging to detect using other techniques. However, its applicability is currently limited by high costs, the need for specialized equipment and trained personnel, as well as the necessity of integrating it with other technologies to obtain a complete view of the genome. Furthermore, accessibility in resource-limited settings remains a significant barrier, potentially hindering its integration in routine clinical practice in the short term.

## 4. Materials and Methods

### 4.1. Optical Genome Mapping

For OGM ultra-high molecular weight, DNA from peripheral blood samples was extracted, digested, and labeled following the manufacturer’s protocols (Bionano Genomics, Inc., San Diego, CA, USA). Labeled DNA was loaded on a Saphyr chip and run on a Saphyr instrument (Bionano Genomics, Inc., San Diego, CA, USA). The *de novo* genome map assembly was performed using BionanoSolve, and SVs were called against the human reference GRCh37 assembly. Data were analyzed with Bionano Access and Bionano Tools on Saphyr Compute Servers (Bionano Genomics, Inc., San Diego, CA, USA). A molecule quality report was generated for each data set and included three key metrics to evaluate sample QC: molecule N50 (≥150 kbp), map rate, and effective coverage. Molecule N50 is a weighted average of all molecules according to their sequence length and is used herein to assess the size distribution of DNA ≥ 150 kbp. The map rate metric is calculated as the fraction of the DNA molecules aligned to GRCh38. The effective coverage is calculated by the coverage depth of molecules aligned to the reference genome (GRCh38). Analytical QC targets were set to achieve 230 kbp of molecule N50 (≥150 kbp), ≥70% map rate, and ≥160X effective coverage of the genome.

### 4.2. Exome Sequencing

Patient and parental genomic DNA were obtained from peripheral blood leukocytes using standard methods. Exome sequencing was performed using the SureSelect Human All Exon V8 (Agilent Technologies, Santa Clara, CA, USA) and run on the Illumina NextSeq500 platform following the manufacturer’s protocol to obtain a minimum reading depth of 100X. Read alignments, variant calling, and annotations were performed on the Alissa Interpret platform (Agilent Technologies). Disease-causing genes related to neurodevelopmental disorders and candidate genes reported in different databases were analyzed. To evaluate the clinical impact and to assess the pathogenicity of the variants, previously reported criteria were used [20,21].

## 5. Conclusions

This study highlights the transformative potential of OGM in resolving elusive genetic etiologies in congenital rare diseases. In Patient 1, OGM precisely delineated a translocation disrupting *BCL11A*, linking it to Dias-Logan syndrome and extending the understanding of its phenotypic spectrum. In Patient 2, OGM identified a mosaic deletion proximal to the *IHH* gene, causing ectopic regulatory interactions and a phenotype resembling acrocallosal syndrome. These findings validate the utility of OGM in detecting SVs overlooked by conventional methods, providing crucial insights into the complex genomic architecture underlying rare disorders. Based on these illustrative examples, we propose that integrating OGM into routine diagnostic workflows will significantly enhance genetic diagnosis, facilitate the discovery of previously unidentified syndromes, and eventually improve the clinical management of patients with rare diseases through advanced personalized medicine.

## Data Availability

Data are contained within this article.

## Abbreviations

The following abbreviations are used in this manuscript:

CGH-Array	Array comparative genomic hybridization
HbF	Fetal hemoglobin
OGM	Optical genome mapping
RDs	Rare diseases
SVs	Structural variants
TADs	Topologically Associated Domains
